# Study-Aware Meta-Analysis Reveals a Recurrent Proteostasis Program and Context-Dependent Gene-Level Responses in Bovine Heat-Stress Transcriptomes

**DOI:** 10.3390/genes17080946

**Published:** 2026-08-13

**Authors:** Xiaotong Zhao, Hua Chang, Quanpeng Zhang, Zhuoyu Zhao, Sihui He, Zongyan Lu, Xun Xiang

**Affiliations:** 1 College of Animal Science and Technology, Yunnan Agricultural University, Kunming 650201, China; xiaotongzhao79@gmail.com (X.Z.); 18591086151@163.com (Z.Z.); 1320780875sh@sina.com (S.H.); 14736845802@163.com (Z.L.); 2 College of Veterinary Medicine, Yunnan Agricultural University, Kunming 650201, China; changhuaxx@126.com; 3 Kunming Animal Health Supervision Institute, Kunming 650041, China; 20011752@163.com

**Keywords:** cattle, heat stress, RNA sequencing, random-effects meta-analysis, modified Knapp–Hartung, tissue sensitivity, gene-set enrichment, single-nucleus RNA sequencing

## Abstract

**Background/Objectives**: Bovine heat-stress RNA-seq studies differ in tissue, age, physiological state, and exposure design. We asked which responses recur across these contexts and which depend on the evidence base. **Methods**: We reprocessed 107 libraries from five in vivo *Bos taurus* studies and synthesized within-study heat-minus-control log_2_ fold changes using restricted maximum-likelihood random-effects models with modified Knapp–Hartung inference. Eight tissue- and age-aware scenarios tested the cross-context estimate. A six-component heat-stress transcriptomic stability index (HSTSI) was benchmarked against five simpler rankings, and an independent mammary single-nucleus dataset provided cell-resolved comparison. **Results**: Of 266 FDR-significant pathways, 259 retained direction across all five study deletions and 21 remained significant in every deletion. Translation, ribosome, protein folding, endoplasmic-reticulum processing, proteasome, and heat-response programs formed the most recurrent axis. Four principal sensitivity scenarios retained 0.920–0.932 gene-direction agreement and 0.957–0.981 effect-rank correlation with the five-study analysis. In the single-nucleus dataset, leading-edge genes from the principal proteostasis programs showed 89.4–100% pooled-nucleus direction agreement and 94.7–100% agreement among published cluster-level differentially expressed genes. HSTSI had the highest mean top-200 held-out direction agreement (0.583 versus 0.516–0.562), with variation among folds. None of 16,756 genes met a modified Knapp–Hartung FDR below 0.10. *IL1R2* and *SDCBP2* were externally concordant, whereas *GZMK* and *CD8A* were context-dependent. **Conclusions**: A coordinated proteostasis program was the most transferable heat-stress signal. Energy remodeling, immune-associated bulk signals, and individual genes showed greater context dependence and define priorities for tissue-matched follow-up.

## 1. Introduction

Heat stress impairs cattle production, inflammatory control, disease resistance, and welfare [1,2,3]. Environmental and nutritional interventions can reduce exposure, while genetic selection and molecular phenotyping may support longer-term thermotolerance. Because the trait is polygenic and biologically distributed, molecular candidates require evidence beyond a single tissue, exposure protocol, or processing strategy [4,5].

Bulk RNA sequencing has resolved different parts of the bovine heat-stress response in distinct experimental settings. Mammary studies have described heat-shock, involution, metabolic, and milk-synthesis programs [6,7,8]. Adult and pre-weaning liver studies have implicated mitochondrial metabolism, protein folding, inflammation, and nutrient partitioning [9,10]. Skin transcriptomics has linked thermal exposure to local metabolic, antioxidant, and immune responses [11], while blood and multi-omics studies have extended the response to systemic immunity, metabolism, and intergenerational exposure [12,13,14]. A recent single-nucleus analysis of heat-stressed bovine mammary gland further showed that proteostasis, cell state, and signaling responses differ among epithelial, stromal, and immune populations [15].

These studies differ in tissue, developmental stage, physiological state, breed, exposure duration, annotation, filtering, and statistical power. Bulk tissue also combines changes in cell abundance and transcriptional state. Comparing published differentially expressed gene lists cannot separate these sources because list membership depends on both effect size and precision. Cross-study synthesis must instead retain each original contrast, place effects on a common scale, and define the biological meaning of their average.

Only a few eligible in vivo studies are available. Standard random-effects models estimate the mean and between-study variance, but normal-approximation intervals can be too narrow when study numbers are small and precisions differ. Modified Knapp–Hartung inference uses a t reference distribution and prevents its adjusted standard error from falling below the conventional value [16]. Prediction intervals estimate the range expected in a comparable future context [17]. Both are relevant when thousands of genes are tested with only two to five contributing studies.

We therefore tested reproducibility at gene, pathway, and cell-class levels. Five datasets were uniformly reprocessed, but heat-minus-control effects were estimated within each original design. The primary analysis estimated the cross-context average; tissue- and age-aware scenarios measured its dependence on study composition. HSTSI ranked genes by six evidence components and was compared with established alternatives in held-out studies. Directional enrichment and an independent single-nucleus dataset were then used to test whether coordinated programs transferred more consistently than individual genes.

## 2. Materials and Methods

### 2.1. Dataset Search, Eligibility, and Contrast Definition

NCBI GEO/DataSets, NCBI SRA, and EBI BioStudies were searched through 11 June 2026. Search terms combined heat stress, thermal stress, heat load, high temperature, or hyperthermia with *Bos taurus*, cattle, bovine, or dairy cow, together with RNA-seq or transcriptome terms. After title and metadata deduplication, 23 plausible GEO series underwent eligibility assessment; five were included, and 18 were excluded. Primary-analysis eligibility required an in vivo *B. taurus* bulk RNA-seq experiment, contemporaneous heat-stress and non-heat comparison groups, at least three biological units per group, and publicly accessible raw reads. In vitro, organoid, single-cell or single-nucleus, developmental-only, technical library-preparation, and longitudinal before–after studies without a concurrent non-heat group were excluded. Complete search strings, candidate records, evidence links, and final decisions are provided in Supplementary Table S1.

Five studies met these criteria: GSE108840, GSE226351, GSE227323, GSE244620, and GSE289946 (Table 1). Contrasts were defined from GEO SOFT records, SRA run metadata, and the corresponding articles before differential-expression analysis [6,9,10,11,12]. GSE108840 contributed 48 post-dry-off mammary libraries from 12 cows and retained time in the model. GSE226351 contributed thermoneutral and heat-stressed liver groups; its pair-fed group was excluded. GSE227323 contributed postnatally cooled and heat-stressed calf liver samples. GSE244620 contributed control and heat-stressed skin samples. Because its sample-title suffixes conflicted with the GEO case/control characteristics, treatment assignment followed the provider matrix and was checked against the published genome-wide effects. GSE289946 contributed dam whole-blood samples; offspring and samples without heat-stress information were excluded. The primary dataset contained 107 libraries (Supplementary Tables S2 and S3); the GSE244620 direction check is documented in Supplementary File.

### 2.2. Read Processing and Transcript Quantification

FASTQ files were checked against the provider-supplied integrity information and evaluated with FastQC 0.12.1 and MultiQC 1.35. Adapter and quality trimming used Trim Galore 2.2.0 with a Phred threshold of 20 and a minimum retained length of 36 nucleotides. All studies were quantified against the Ensembl release 110 *B. taurus* ARS-UCD1.2 cDNA reference. Salmon quantification used automatic library-type detection, selective alignment validation, sequence-bias correction, and GC-bias correction [18]. Both single-end and paired-end libraries were handled according to their run metadata; matrix-concordance results are reported in Supplementary Table S16.

### 2.3. Gene-Level Import and Study-Specific Differential Expression

Transcript estimates were summarized to Ensembl gene identifiers with tximport 1.38.2 using type = salmon, ignoreTxVersion = TRUE, and countsFromAbundance = no [19]. This passes estimated counts and effective lengths to DESeq2 so that gene-level offsets retain transcript-length information. Concordance with a parallel transcript-to-gene aggregation was evaluated using library-level Pearson correlations and median absolute log-scale differences.

Differential expression was estimated separately for each study with DESeq2 1.50.2 [20]. Genes required counts of at least 10 in at least 20% of the study libraries. GSE108840 used ~time + analysis_group; the other studies used ~analysis_group. Unshrunken maximum-likelihood log_2_ fold changes and Wald standard errors were used because random-effects synthesis requires likelihood-based estimates and sampling errors. Coefficients were oriented as heat relative to control.

### 2.4. Random-Effects Meta-Analysis and Small-Sample Inference

For gene g in study i, the DESeq2 log_2_ fold change y_gi_ and its standard error s_gi_ were entered into a univariate random-effects model,

$$y_{gi}=\mu_{g}+u_{gi}+\varepsilon_{gi},\;\;u_{gi}∼N(0,\tau_{g}^{2}),\;\varepsilon_{gi}∼N(0,s_{gi}^{2}),$$

where *μ_g_* is the mean cross-study effect, and *_τ_^g^*^2^ is the between-study variance. Models were fitted by REML with metafor 5.0-1 [21]. A gene required finite effects and positive standard errors in at least two studies. Conventional REML standard errors and normal-approximation tests were retained for calibration comparisons.

Primary inference used a modified Knapp–Hartung standard error. For each gene, the standard error was

$$s_{g,mKH}=max(s_{g,REML},s_{g,KH}),$$

and two-sided tests and 95% confidence intervals used a t-distribution with k_g_-1 degrees of freedom [16]. The primary plug-in 95% prediction interval was

$${\hat{\mu }}_{g}\pm t_{0.975,k_{g}-1}\sqrt{s_{g,mKH}^{2}+{\hat{\tau }}_{g}^{2}}.$$

Because prediction-interval conventions are unstable when k is small, classification was also repeated for genes with k_g_ ≥ 3 using the more conservative t_0.975,kg_-2 critical value. Benjamini–Hochberg correction was applied across all genes with estimable meta-analytic effects [22]. I^2^, τ^2^, and the Cochran Q test were recorded as heterogeneity descriptors. Models that did not converge under default Fisher scoring were refitted with a step adjustment of 0.5, a maximum of 10,000 iterations, and a convergence threshold of 10^−8^. Persistent non-convergence was prespecified as an exclusion criterion. Fisher and signed Stouffer combinations of study-level *p*-values were secondary diagnostics, while inferential conclusions were based on the effect-size model. Complete gene-level results and prediction-interval sensitivity are supplied in Supplementary Tables S5 and S18.

### 2.5. Cross-Context Estimand and Tissue-Aware Sensitivity Analyses

The primary estimand was the mean heat-minus-control effect across the eligible in vivo bulk-tissue contrasts. Each study retained its original biological unit and design. Restricted and cell-resolved analyses addressed tissue-specific questions.

Eight sensitivity scenarios altered study composition. Solid-tissue, adult-only, and adult-solid analyses excluded whole blood, pre-weaning calf liver, or both. Further scenarios excluded mammary gland, adult liver, both liver studies, or skin; a descriptive liver-only analysis combined the two liver studies. Each scenario repeated the REML, modified Knapp–Hartung, prediction-interval, and FDR procedures. Agreement with the primary analysis was measured by effect-direction concordance and Spearman correlation; the two liver studies were also compared directly. Full definitions and candidate estimates are in Supplementary Tables S25 and S26.

### 2.6. HSTSI Construction and Sensitivity Analysis

HSTSI was calculated for every gene with an estimable random-effects model. Six components were scaled to [0, 1]: modified Knapp–Hartung FDR support, S_q_ = min[-log_10_(q_mKH_)/10, 1]; effect magnitude, S_β_ = min(|μ|, 1); study coverage, S_k_ = min(k/5, 1); direction consistency, S_d_ = max(n_up_, n_down_)/(n_up_ + n_down_); low heterogeneity, S_h_ = 1 − I^2^/100 after bounding I^2^ to [0, 100]; and jackknife support, S_j_ = 0.7p_same direction_ + 0.3p_P_mKH_ < 0.10. Missing I^2^ was assigned 0.5, and unavailable jackknife support was assigned zero. The score was

$$HSTSI=100(0.25S_{q}+0.20S_{\beta }+0.20S_{k}+0.15S_{d}+0.10S_{h}+0.10S_{j}).$$

HSTSI served exclusively as a prioritization score; formal statistical inference remained based on the random-effects model.

For genes represented in at least three studies, the gene-level jackknife removed each available study effect in turn and contributed only to S_j_. Weight sensitivity used 500 perturbations with random seed 20,260,710; each base weight was multiplied by an independent value from a uniform 0.75–1.25 distribution and then renormalized. We recorded top-200 frequency and the rank distribution for each gene. Definition sensitivity compared the base score with nine alternatives: equal component weights, six leave-one-component-out scores, a hyperbolic-tangent transformation of effect magnitude, and an effect cap of two rather than one. Rank correlations and overlaps across the top 100–500 genes were calculated for each alternative.

Separately, five complete leave-one-study-out analyses removed each study from the entire analysis and repeated random-effects fitting, modified Knapp–Hartung inference, multiple-testing correction, the nested gene-level jackknife component, and HSTSI ranking. Stability was summarized by top-200 overlap and Jaccard index, Spearman rank correlations for all genes and the full-data top 200, direction agreement, and candidate-specific rank distributions. Tier A required effects from all five studies, direction consistency of at least 0.80, and complete jackknife direction agreement. It also required weight-perturbation and leave-one-study-out top-200 frequencies of at least 0.80. Tier B required effects from at least four studies, direction consistency at least 0.75, jackknife direction agreement at least 0.80, weight frequency at least 0.50, and leave-one-study-out frequency at least 0.60. The remaining genes in the top 200 were assigned Tier C. Statistical significance and heterogeneity were reported separately from these ranking-stability tiers. Complete HSTSI, top-200, per-study, jackknife, and scenario results are provided in Supplementary Tables S6–S10 and S19–S21.

### 2.7. Held-Out-Study Benchmark of Candidate-Ranking Methods

HSTSI was compared with rankings based on the absolute pooled effect, the modified Knapp–Hartung *p*-value, the prediction-interval margin, the Fisher-combined *p*-value, and signed Stouffer *p*-value. In each outer fold, one study was withheld; all rankings, including the nested HSTSI jackknife, were reconstructed from the remaining studies. The held-out study was used only for evaluation. The primary metric was direction agreement among the top 200 genes; top-50, top-100, and top-500 agreement, and held-out z-statistics were secondary metrics (Supplementary Table S27).

### 2.8. Directional Functional Enrichment

Ensembl identifiers were mapped to bovine Entrez identifiers with org.Bt.eg.db 3.22.0 and GO.db 3.22.0. The directional ranking statistic was μ_g_/s_g,mKH_; duplicate Entrez mappings retained the entry with the largest absolute statistic. Gene-set enrichment analysis (GSEA) used the complete ranking, gene sets of 10–500 genes, and fgsea 1.36.2 through clusterProfiler 4.18.4 [23,24]. Bovine KEGG tables were cached on 10 July 2026. Direction-stratified over-representation analysis (ORA) used mapped top-200 genes and all tested bovine Entrez genes as the universe. Benjamini–Hochberg correction was applied within each enrichment family.

GO and KEGG GSEA were repeated after each study deletion and in every tissue- or age-aware scenario. For each full-data FDR-significant term, we recorded direction and FDR recurrence. The primary, deletion, and grouped analyses used fixed scenario-specific seeds and the same software and annotation resources. Resource checksums and session information are in Supplementary File; complete results are in Supplementary Tables S11–S15, S23, S24, and S29.

### 2.9. Independent Cell-Resolved Comparison

GSE317482 provided an external bovine mammary single-nucleus RNA-seq comparison [15]. The source study included thermoneutral and heat-stressed late-lactation cows (*n* = 3 per condition), but biopsies were pooled within treatment before library preparation; condition contrasts were therefore descriptive.

For the pooled comparison, TN and HS counts were summed across nuclei, converted to CPM, and expressed as log_2_[(CPM_HS_ + 0.1)/(CPM_TN_ + 0.1)]. Genes required at least 10 counts and detection in at least 10 nuclei. The published significant cluster-level table was separately reoriented as heat stress minus thermoneutral and summarized by median direction across significant clusters. Agreement was evaluated for the four candidates and leading-edge genes from seven representative programs (Supplementary Table S28).

For atlas reconstruction, nuclei required 1000–40,000 total counts, 200–2500 detected genes, and <10% counts assigned to *MT-*genes; genes detected in fewer than three nuclei were removed. Counts were normalized to 10,000 per nucleus and transformed with log(1 + x). Five thousand highly variable genes and 30 principal components were used for Harmony integration, a 20-nearest-neighbor graph, Leiden clustering, and UMAP. Harmony-corrected components were used only for graph construction, clustering, and visualization; expression summaries used the uncorrected log-normalized matrix. Leiden resolution 0.8 was selected by a prespecified screen, and clusters were assigned to 10 broad epithelial, stromal, and immune classes using source-study markers. Candidate panels summarized detection rate and mean expression; program panels summarized the HS–TN difference in mean standardized leading-edge scores. Cluster correspondence and source data are provided with Supplementary Table S28 and the accompanying files.

### 2.10. Numerical Validation and Quality Control

An independent implementation refitted all 16,756 gene-level random-effects models directly from the study-effect table. Estimates, heterogeneity statistics, confidence and prediction intervals, and adjusted probabilities were compared with the primary analysis. HSTSI values, prediction-interval classifications, study-deletion summaries, enrichment directions, and pathway recurrence were also reconstructed from their component tables. Software session information and the validation criteria accompany the derived results (Supplementary Tables S17 and S22).

## 3. Results

### 3.1. Five Studies Defined a Cross-Context Bulk-Tissue Estimand

**Figure 1:**
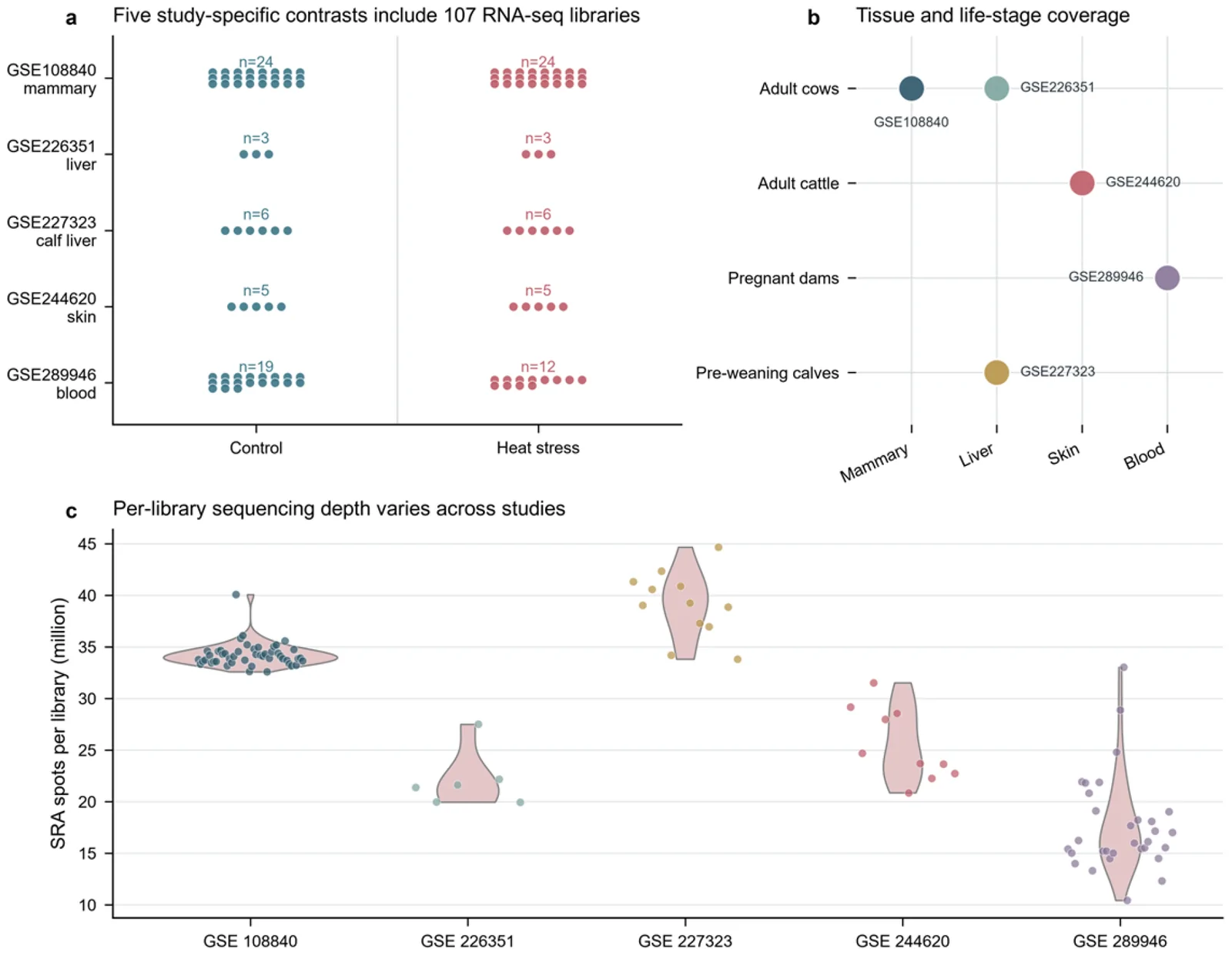
Overview of the five-study analysis. (**a**) Control and heat-stress libraries by study; points denote libraries and labels give counts. (**b**) Tissue and life-stage coverage; symbols denote studies. (**c**) SRA spot-count distributions by study. Differential expression was estimated within study before integration. Colors distinguish conditions or studies, points show individual libraries, circles denote studies, and violins summarize study-level distributions.

The analysis included 107 mammary, liver, skin, and whole-blood libraries (Figure 1). Before study-specific filtering, tximport produced 22,372 gene rows per study and closely matched the parallel aggregation (minimum library-level correlation, 0.999973; median absolute log-scale difference, 0). The five studies tested 13,853–16,760 genes and identified 4–12,116 within-study genes at FDR < 0.05 (Supplementary Figure S1; Supplementary Table S4).

For GSE244620, all 10 libraries matched the provider-matrix groups. Current heat-minus-control effects correlated positively with the published effects (Pearson r = 0.623; Spearman ρ = 0.587). The merged table contained 18,255 genes; 16,756 had effects from at least two studies, and 15,396 had effects from at least three.

### 3.2. Tissue-Aware Sensitivity Varied by Scenario

Solid-tissue, adult-only, adult-solid, and no-liver analyses retained direction agreement of 0.920–0.932 and effect-rank correlations of 0.957–0.981 with the five-study analysis (Supplementary Table S25). Excluding the mammary gland or adult liver yielded correlations of 0.971 and 0.979 and direction agreement of 0.884 and 0.947. No scenario identified a gene at modified Knapp–Hartung FDR < 0.10.

Excluding skin reduced direction agreement to 0.645 and effect-rank correlation to 0.371. Liver-only estimates had a directional agreement of 0.499 and an effect-rank correlation of 0.057 with the primary result; none of the 14,167 genes had a 95% prediction interval excluding zero. The two liver studies themselves had an effect-rank correlation of −0.008 and direction agreement of 0.502.

*IL1R2* and *SDCBP2* remained positive, and *GZMK* and *CD8A* remained negative in every scenario (Supplementary Table S26). In solid tissues, all four directions were preserved, but the prediction intervals for *GZMK* and *CD8A* crossed zero.

### 3.3. Small-Sample Calibration Changed Gene-Level Significance

**Figure 2:**
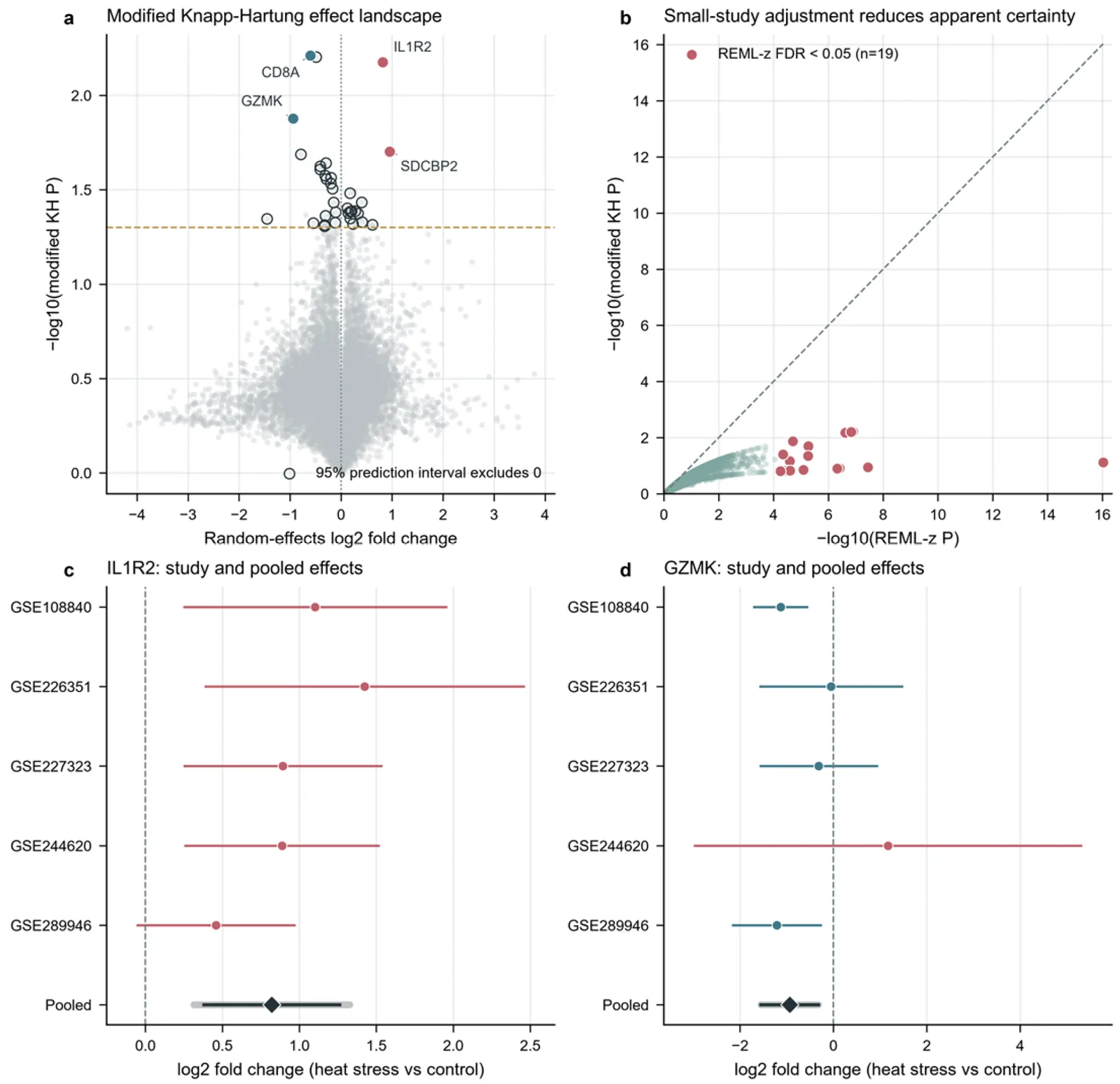
Gene-level inference under conventional and small-sample random-effects models. (**a**) Pooled log_2_ fold change versus unadjusted modified Knapp–Hartung *p*-value for 16,756 genes; open circles indicate 95% prediction intervals excluding zero, and labels mark four candidates. The dashed line is *p* = 0.05. (**b**) Conventional REML-z versus modified Knapp–Hartung *p*-values; highlighted genes have REML-z FDR < 0.05. (**c**,**d**) Study and pooled estimates for *IL1R2* and *GZMK*; intervals show study 95% Wald intervals, pooled 95% confidence intervals, and 95% prediction intervals. Gray points represent all genes; red and teal encode positive and negative effects, open circles mark prediction intervals excluding zero, horizontal lines show confidence intervals, black diamonds show pooled effects, and the dashed line denotes equality.

Conventional normal-approximation tests identified 19 genes at FDR < 0.05, whereas modified Knapp–Hartung inference identified none at FDR < 0.10 (Figure 2a,b). Thirty-four primary prediction intervals excluded zero; 15 did so with the alternative t_k−2_ critical value, and 23 of the 34 primary exclusions had τ^2^ = 0. An independent refit reproduced all gene-level estimates and statistics within 1.85 × 10^−13^.

All five *IL1R2* estimates were positive; its pooled log_2_ fold change was 0.820 (95% confidence interval, 0.379–1.261; prediction interval, 0.315–1.326; I^2^ = 6.1%; Figure 2c). Its unadjusted modified Knapp–Hartung *p*-value was 0.00668, and its FDR was 0.802. *GZMK* had a pooled log_2_ fold change of −0.937 (95% confidence and prediction interval, −1.552 to −0.323; I^2^ = 0%), with one imprecise positive estimate and four non-positive estimates (Figure 2d).

### 3.4. HSTSI Ranked Candidates and Was Benchmarked in Held-Out Studies

**Figure 3:**
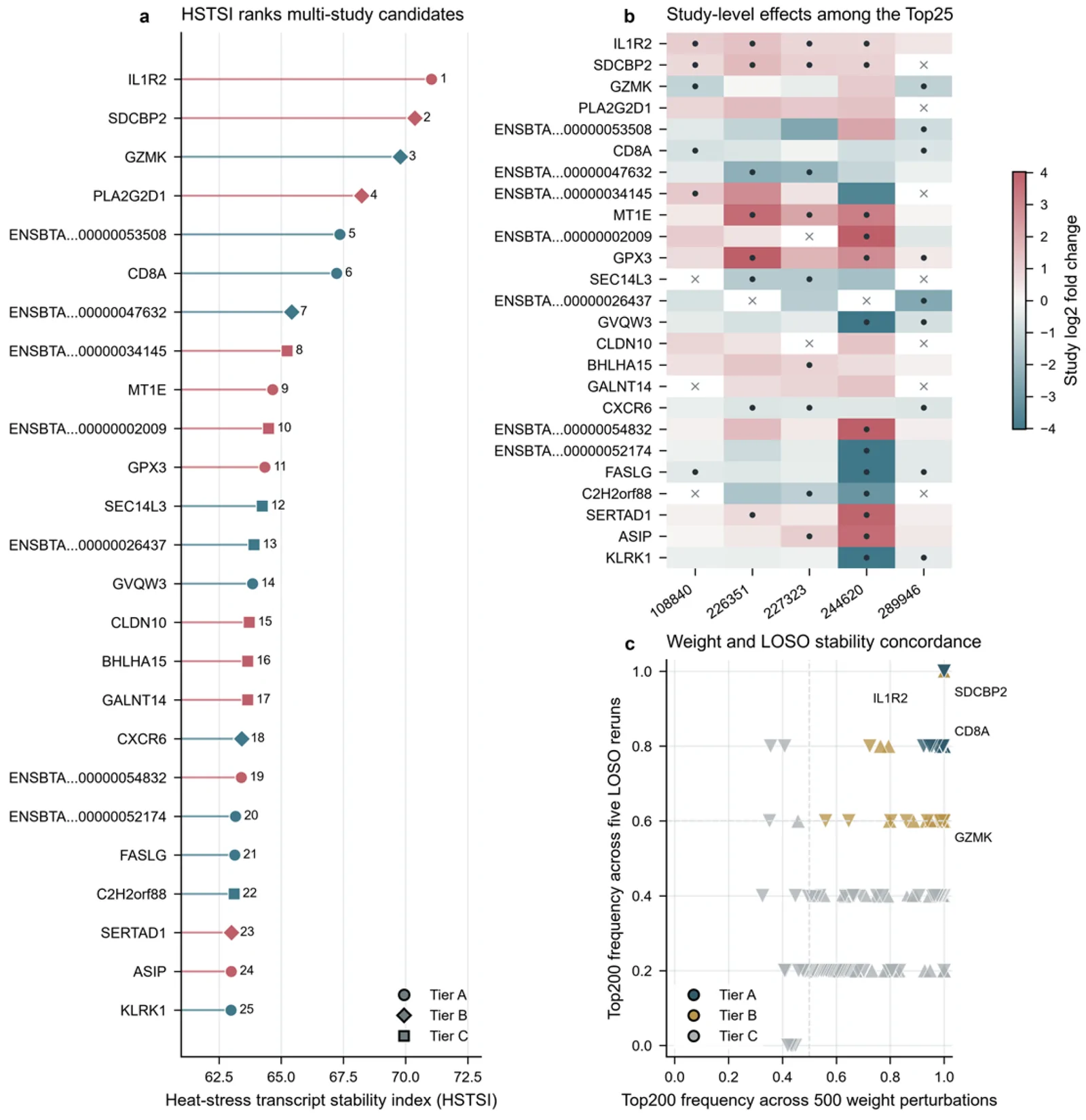
HSTSI candidate prioritization and sensitivity. (**a**) Top 25 candidates, with rank, direction, and stability tier. (**b**) Their study-specific log_2_ fold changes; dots mark nominal *p* < 0.05, and crosses mark unavailable effects. (**c**) Top-200 frequency across 500 weight perturbations and five complete leave-one-study-out analyses. Dashed lines mark 0.50 and 0.60. HSTSI is a prioritization score, not a significance test. Red and teal denote positive and negative pooled effects; marker shape denotes tier, and triangle orientation denotes pooled-effect direction.

HSTSI ranked 16,756 genes using six predefined components (Figure 3a). The top 200 included 67 positive and 133 negative effects: 43 genes met Tier A criteria, 31 met Tier B criteria, and 126 met Tier C criteria (Supplementary Figure S2c).

*IL1R2* ranked first (HSTSI 71.04, Tier A) and remained in the top 200 in all perturbations and deletions (worst deletion rank, 4). *SDCBP2* ranked second (70.37, Tier B) and also remained in every top 200 (worst rank, 12). *GZMK* ranked third (69.79, Tier B) but had a deletion top-200 frequency of 0.60 and a worst rank of 628. *CD8A* ranked sixth (67.23, Tier A), with a deletion top-200 frequency of 0.80 (Figure 3b,c; Supplementary Figure S3a,b).

Across nine alternative definitions, correlation with the base ranking ranged from 0.505 to 1.000 and top-200 overlap from 24 to 200 (Supplementary Figure S4). Removing FDR support left the top 200 unchanged, whereas removing effect magnitude retained 24 genes. *IL1R2*, *SDCBP2*, and *GZMK* remained on all ten top-200 lists, and *CD8A* on nine. By comparison, *HSPA1A*, *HSPB1*, and *HSP90AA1* ranked 935th, 1883rd, and 4442nd and had I^2^ values of 90.5–98.5%.

Mean top-200 held-out direction agreement was 0.583 for HSTSI, 0.562 for Fisher, 0.556 for signed Stouffer, 0.543 for modified Knapp–Hartung *p*-value, and 0.516 for both absolute effect and prediction-interval margin (held-out benchmark; Supplementary Table S27). HSTSI fold values were 0.745, 0.640, 0.400, 0.575, and 0.555 for GSE108840, GSE226351, GSE227323, GSE244620, and GSE289946, respectively. The lowest value (0.400) occurred when GSE227323 was held out; this study was the only pre-weaning calf-liver context in the evidence base.

### 3.5. Complete Study Deletion Preserved Direction More Strongly than Rank

**Figure 4:**
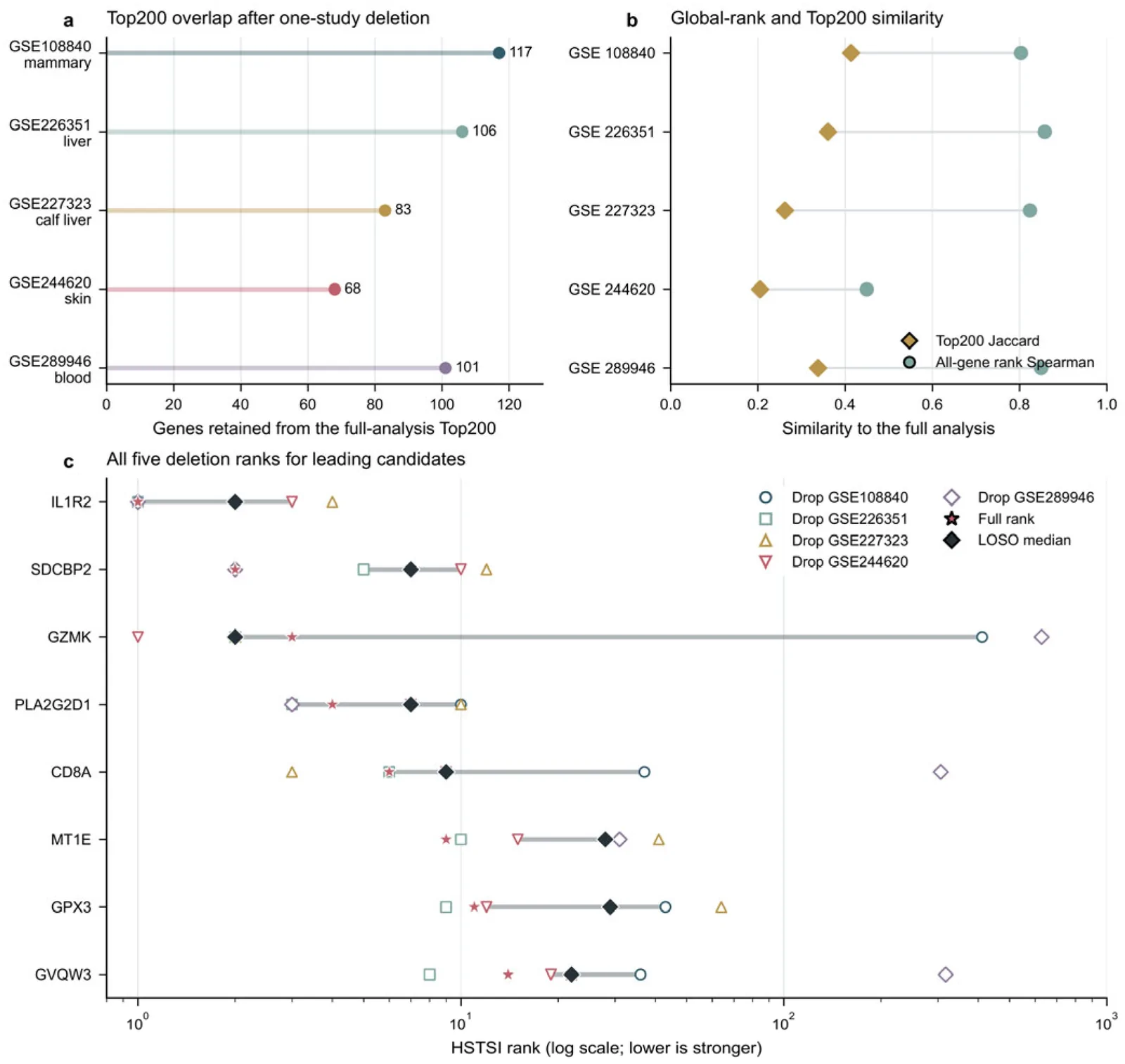
Five complete leave-one-study-out analyses. (**a**) Overlap between each deletion-specific and full-data top 200. (**b**) Top-200 Jaccard index and all-gene rank correlation with the full analysis. (**c**) Full-data and deletion-specific candidate ranks. Each deletion repeated model fitting, inference, multiple-testing correction, nested jackknife scoring, and HSTSI ranking.

Top-200 overlap after individual study deletion was 117, 106, 83, 68, and 101 for GSE108840, GSE226351, GSE227323, GSE244620, and GSE289946, respectively (Figure 4). Corresponding Jaccard indices were 0.413, 0.361, 0.262, 0.205, and 0.338; all-gene rank correlations ranged from 0.449 to 0.858. Direction agreement for the full-data top 200 was 1.00 in every deletion, while median deletion ranks ranged from 169.5 to 461.5 (Supplementary Figure S3c).

*IL1R2* and *SDCBP2* remained near the top in all deletions. *GZMK* and *CD8A* preserved their negative direction but moved outside the top 200 in some deletions.

### 3.6. Pathway Directions Were Stable Across Study Deletions

**Figure 5:**
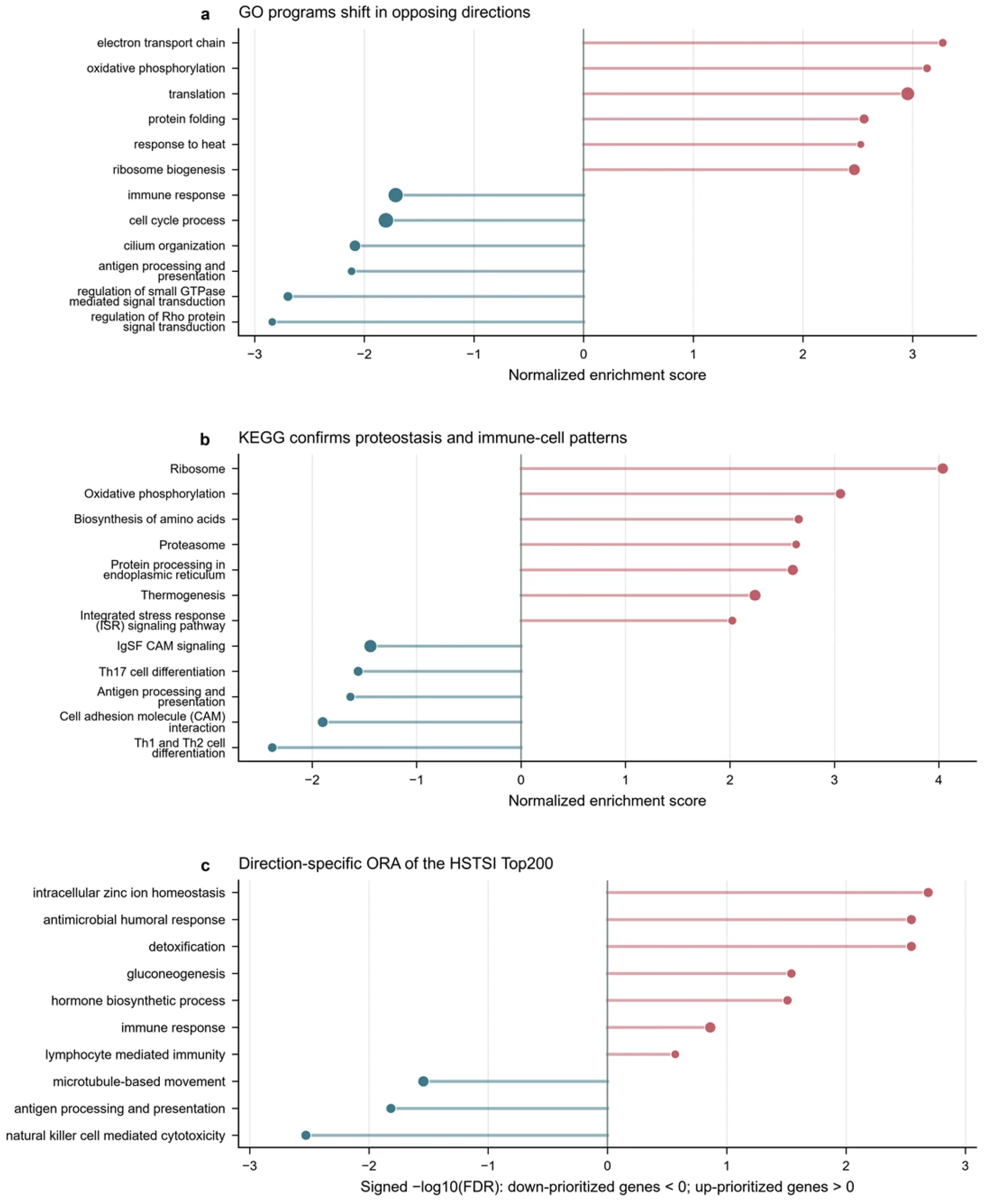
Directional functional programs. (**a**,**b**) Representative GO and bovine KEGG GSEA terms; position denotes normalized enrichment score, color denotes direction, and point size denotes gene-set size. (**c**) Direction-stratified GO ORA of the mapped HSTSI top 200; position is signed −log_10_(FDR) and point size denotes mapped genes.

The meta-analytic ranking mapped to 14,408 bovine Entrez identifiers. GSEA identified 215 GO biological processes and 51 KEGG pathways at FDR < 0.05 (Figure 5a,b). Positive programs included translation (NES 2.96, FDR 3.06 × 10^−19^) and protein folding (NES 2.56, FDR 1.07 × 10^−6^), together with ribosome, oxidative phosphorylation, proteasome, endoplasmic reticulum processing, thermogenesis, and response to heat. Negative programs included small GTPase regulation (NES −2.70, FDR 5.64 × 10^−8^) and antigen processing and presentation (NES −2.12, FDR 0.00471), together with T-cell receptor signaling, leukocyte cytotoxicity, cell cycle, Th1/Th2 differentiation, and cell adhesion.

Among 128 mapped genes from the HSTSI top 200, ORA associated up-prioritized genes with zinc-ion homeostasis, detoxification, humoral defense, gluconeogenesis, and hormone biosynthesis. Down-prioritized genes were associated with lymphocyte and natural killer cell immunity, cytotoxicity, antigen presentation, adhesion, and the cell cycle (Figure 5c). Complete GSEA and ORA results are in Supplementary Tables S11–S14.

Of the 266 full-data FDR-significant pathways, 259 retained direction in all five study deletions and seven did so in four. Full-data NES correlated with median deletion-specific NES at 0.972, and 21 pathways remained significant in every deletion (Supplementary Figure S5; Supplementary Tables S23 and S24).

**Figure 6:**
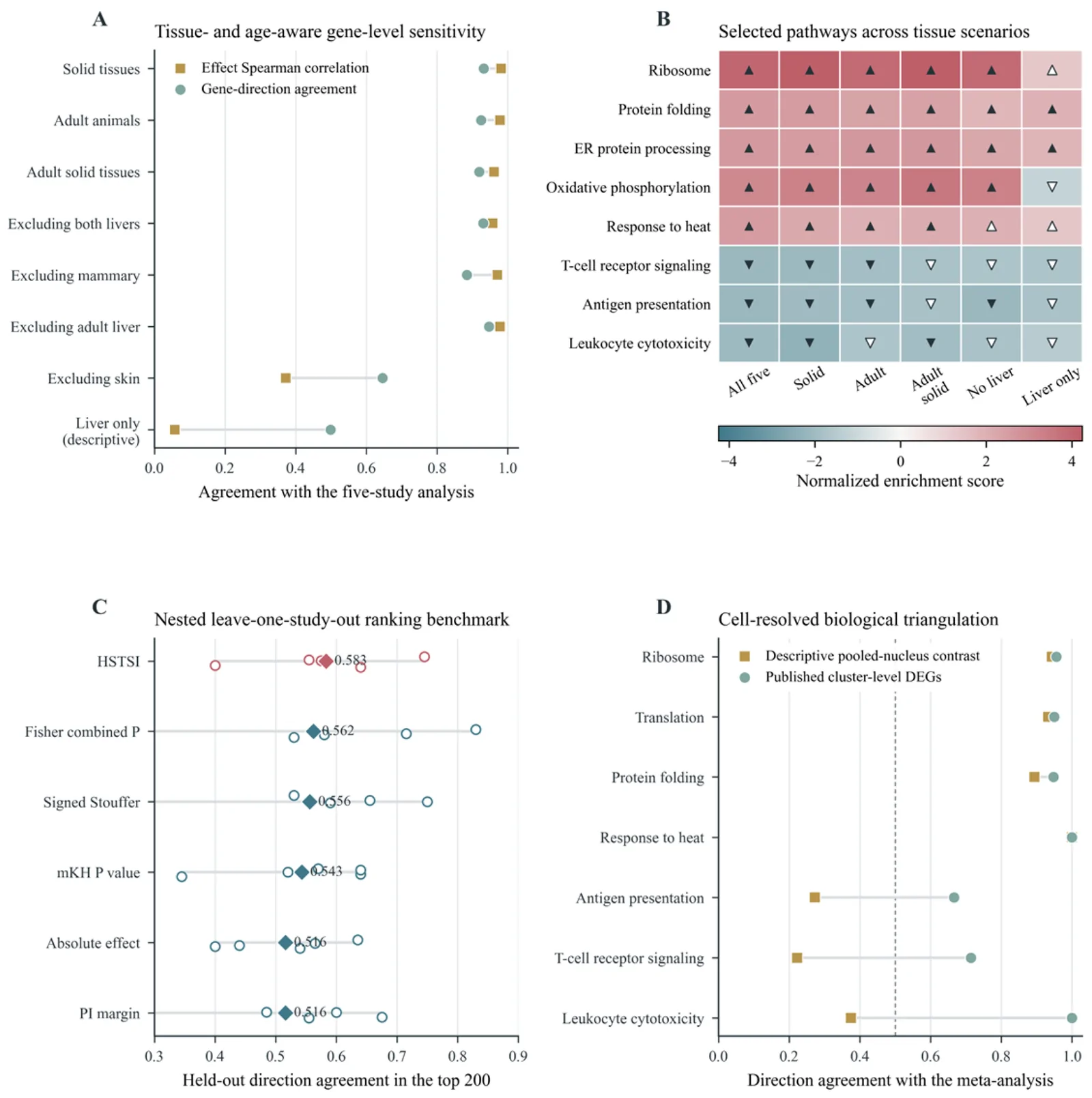
Sensitivity and external comparison. (**A**) Effect-rank correlation and direction agreement by scenario. (**B**) Normalized enrichment scores of selected pathways; triangle direction indicates the sign, and fill indicates FDR < 0.05. (**C**) Held-out top-200 direction agreement. (**D**) Leading-edge gene direction agreement with pooled and cluster-level GSE317482 contrasts; dashed line, 0.50. In panel (**C**), open circles show study-specific held-out values, diamonds show method means, and red highlights HSTSI.

In the 14-term tissue-sensitivity panel, all directions were retained in the solid-tissue, adult-only, adult-solid, adult-liver-deletion, and no-liver analyses (Figure 6B; Supplementary Table S29). Thirteen directions remained after excluding mammary gland or skin. The liver-only analysis retained 10, including positive translation and protein folding, and negative antigen presentation, T-cell receptor, and cytotoxicity programs; oxidative phosphorylation and proteasome changed direction.

### 3.7. Cell-Resolved Comparison Reproduced Proteostasis Directions

GSE317482 reported 14 mammary cell clusters [15]. Pooled-nucleus direction agreement was 94.3% for ribosome, 93.3% for translation, 89.4% for protein folding, and 100% for response to heat (Figure 6D; Supplementary Table S28). Among genes significant in at least one published cluster, agreement was 95.6%, 95.0%, 94.7%, and 100%, respectively.

For antigen processing, T-cell receptor signaling, and leukocyte cytotoxicity, the pooled-nucleus agreement was 27.3%, 22.2%, and 37.5%. The corresponding agreement among significant cluster-level genes was 66.7%, 71.4%, and 100%, respectively; the cytotoxicity value was based on one gene.

*IL1R2* was positive in the pooled contrast but absent from the published significant cluster table. *SDCBP2* was positive in the pooled contrast and in two significant luminal clusters. *GZMK* and *CD8A* were positive in the pooled mammary contrast and absent from the significant cluster table, opposite to their negative meta-analytic directions.

**Figure 7:**
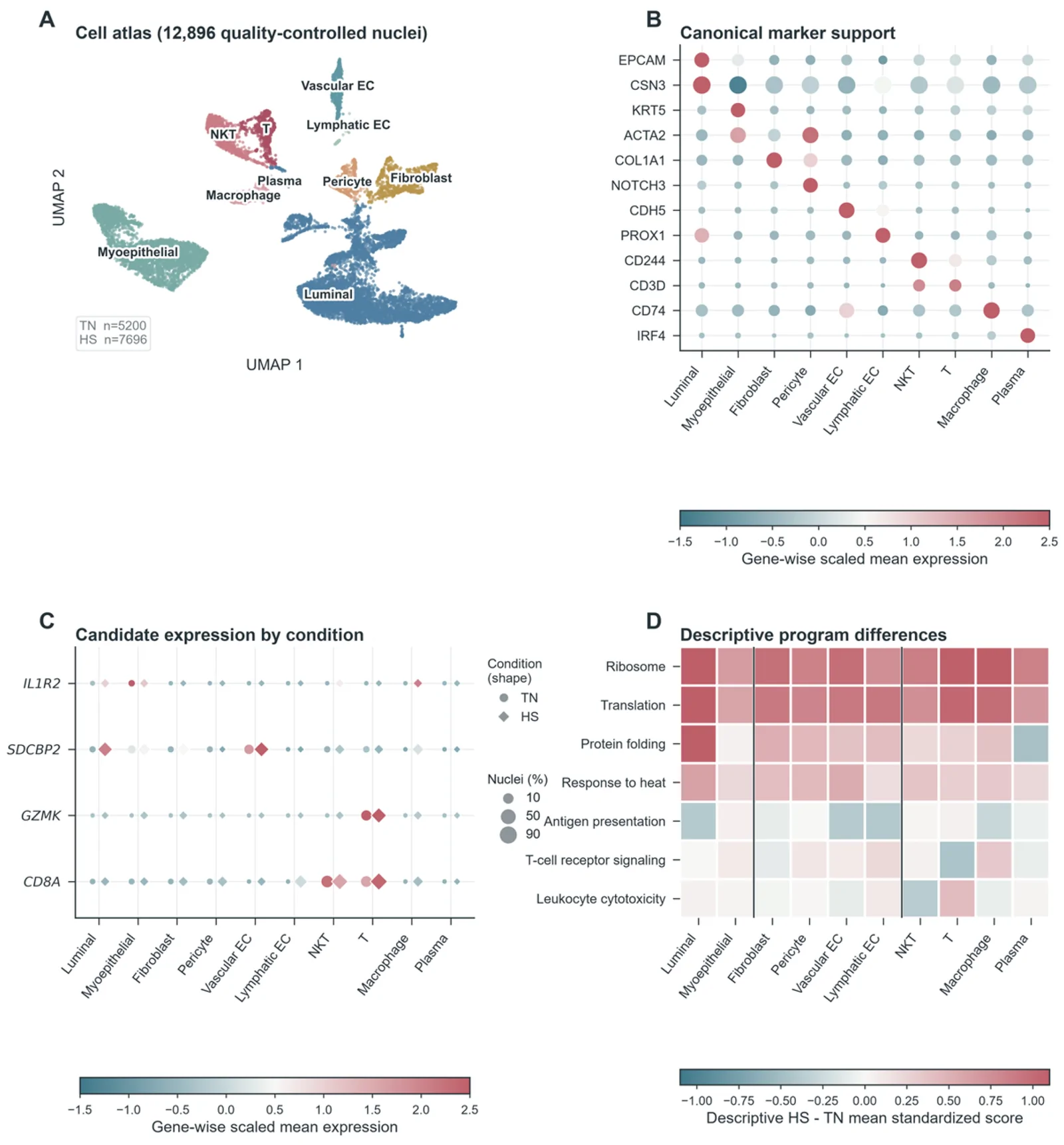
Single-nucleus localization in bovine mammary gland. (**A**) UMAP of 12,896 nuclei in 10 broad classes. (**B**) Marker support; dot size denotes detection rate, and color denotes scaled expression. (**C**) Candidate expression by class and condition. (**D**) Descriptive HS–TN score differences for seven leading-edge programs. Source biopsies were pooled within treatment. In panel (**D**), vertical lines separate epithelial, stromal/endothelial, and immune classes.

Raw-matrix reprocessing retained 5200 TN and 7696 HS nuclei. Twenty-one Leiden clusters were assigned to 10 marker-supported broad classes (Figure 7A,B). Ribosome, translation, and response-to-heat scores were higher under HS in all 10 classes, and protein folding in nine. Immune-program differences varied in direction among cell classes (Figure 7D). *GZMK* and *CD8A* localized mainly to lymphoid compartments; *SDCBP2* was broader and higher under HS in the luminal and vascular endothelial classes, whereas *IL1R2* was sparsely detected (Figure 7C).

## 4. Discussion

The analyses showed that reproducibility depended on biological scale. No gene passed the modified Knapp–Hartung correction across the transcriptome, but coordinated pathways retained direction across study deletion, tissue restriction, and an external cell-resolved comparison. Candidate genes occupied an intermediate level: their directions were often stable, whereas ranks and external support varied. Proteostasis was therefore the most transferable signal, followed by context-dependent energy, immune, and gene-level responses.

The primary model estimates an average across eligible in vivo contexts rather than a tissue-invariant mechanism. Its principal grouped sensitivities closely matched the five-study effects, supporting recurrence across solid tissues and adult animals. Gene ordering was less stable after removal of GSE244620, despite independent confirmation of its treatment direction, and the two liver studies were nearly uncorrelated. Matching tissue alone therefore did not remove differences in age, exposure, and study design. In contrast, pathway direction changed little across the same restrictions, indicating that coordinated programs were more stable than exact gene ranks.

Calibration for small-k-separated effect estimates from statistical certainty. The same study effects yielded 19 normal-approximation findings but no modified Knapp–Hartung FDR findings. This distinction matters when each gene is informed by only two to five studies [16]. Prediction intervals provided a complementary measure of transfer to future contexts [17], but their classification also depended on the small-k convention. HSTSI used these signals for prioritization rather than significance testing. It had the highest mean held-out direction agreement among the six rankings, although Fisher and signed Stouffer were close and fold-level performance varied. The limited contribution of its FDR component and the sensitivity of the broader top 200 favor a continuous, evidence-annotated ranking over a fixed biomarker panel.

The recurrent positive axis centered on protein synthesis and quality control. Translation, ribosome, endoplasmic-reticulum processing, protein folding, proteasome, and heat-response programs remained positive across the main sensitivity analyses. Independent mammary, cell-culture, and myocyte studies report related remodeling [8,25,26]. In GSE317482, the same leading-edge programs were concordant among published clusters; reconstructed scores increased in nearly every broad mammary cell class [15]. Energy metabolism was less uniform: oxidative phosphorylation was positive in the cross-context analysis but changed direction in the liver-only subset, consistent with context-dependent hepatic mitochondrial responses [9].

The negative immune axis requires a bulk-tissue interpretation. T-cell receptor signaling, antigen presentation, cytotoxicity, and adhesion remained negative in the meta-analysis, consistent with reports that heat exposure can combine inflammatory activation with reduced cellular immune competence [2,4,13,27]. In the single-nucleus comparison, pooled agreement was low for these programs, cluster-level agreement was higher, and reconstructed changes varied among cell classes. *GZMK* and *CD8A* were concentrated in lymphoid compartments. The bulk signal can therefore reflect both cellular abundance and within-cell state; tissue-matched cell-resolved data are needed to separate their contributions outside the mammary gland.

External evidence also divided the four leading candidates. *IL1R2* was consistently positive and is compatible with feedback control of IL-1 signaling [28]. *SDCBP2* was positive in the pooled comparison and two luminal clusters. By contrast, mammary *GZMK* and *CD8A* directions differed from their cross-context estimates, consistent with their dependence on lymphocyte abundance or state [29]. The first two genes form the more externally concordant tier; the latter two require tissue-specific validation.

Unlike conventional livestock meta-analyses of phenotypic outcomes, this study reconstructed thousands of transcript-level effects from uniformly processed reads while preserving each original contrast and its uncertainty [1]. It also extends candidate-gene and GWAS reviews by testing effect recurrence directly rather than cataloging reported loci [3,5]. The resulting framework links average-effect inference, ranking stability, pathway recurrence, tissue sensitivity, and cell-resolved support. Animal-level cohorts with matched cell composition and thermotolerance measurements can now test the strongest pathways and candidates for breeding or diagnostic use.

## 5. Conclusions

These findings establish a practical principle for bovine heat-stress genomics: reproducibility is strongest at the level of coordinated biological programs rather than universal individual-gene markers. By treating tissue, age, and exposure design as explicit biological contexts, study-aware meta-analysis separates transferable proteostasis responses from context-dependent metabolic, immune, and gene-level signals. This distinction supports the selection of broadly relevant pathways while requiring tissue-matched validation of individual biomarkers, and it provides a general framework for integrating heterogeneous livestock transcriptomic evidence.

## Figures and Tables

**Table 1:** Public bovine heat-stress RNA-seq studies included in the primary meta-analysis. Biological-unit and library counts are shown separately.

Dataset	Tissue and Context	Effect Definition	Biological Units	Libraries	Study Model
GSE108840	Mammary gland; dry-period Holstein cows	Heat-stressed minus cooled control at post-dry-off days 3, 7, 14, and 25	6 vs. 6 cows	24 vs. 24	~time + analysis_group
GSE226351	Liver; lactating dairy cows	Heat stress minus thermoneutral control; pair-fed animals excluded	3 vs. 3 cows	3 vs. 3	~analysis_group
GSE227323	Liver; pre-weaning Holstein calves	Postnatal heat stress minus postnatal cooling	6 vs. 6 calves	6 vs. 6	~analysis_group
GSE244620	Skin; adult Brangus cattle	Heat-stress cases minus controls	5 vs. 5 cattle	5 vs. 5	~analysis_group
GSE289946	Whole blood; pregnant German Holstein dams	Heat-stressed dams minus dams without heat stress; offspring excluded	19 vs. 12 dams	19 vs. 12	~analysis_group

## Data Availability

All data supporting this study are included in the main text and Supplementary Materials.

## Supplementary Materials

The following supporting information can be downloaded at: https://www.mdpi.com/article/10.3390/genes17080946/s1, Figures S1–S5; Supplementary Tables S1–S24 in the first workbook; Supplementary Tables S25–S29 in a second workbook; 15 companion CSV files containing complete per-study, jackknife, HSTSI-sensitivity, independent-refit, pathway-deletion, tissue-sensitivity, ranking-benchmark, external-triangulation, and single-nucleus atlas source data; and Supplementary File containing the analysis scripts, software-session records, annotation-resource locks, the GSE244620 direction-verification record, and numerical validation reports. Supplementary Table S28 includes the reconstructed-to-published cluster correspondence, and Supplementary Table S29 reports the grouped tissue-sensitivity GSEA.

## Abbreviations

CPM	counts per million
FDR	false-discovery rate
GEO	Gene Expression Omnibus
GO	Gene Ontology
GSEA	gene-set enrichment analysis
HS	heat stress
HSTSI	heat-stress transcriptomic stability index
KEGG	Kyoto Encyclopedia of Genes and Genomes
mKH	modified Knapp–Hartung
NES	normalized enrichment score
ORA	over-representation analysis
REML	restricted maximum likelihood
RNA-seq	RNA sequencing
SRA	Sequence Read Archive
TN	thermoneutral
UMAP	Uniform Manifold Approximation and Projection
